# Elderly people's perceptions on the use of mobile phones to support the self-management of long-term illnesses at Kiruddu National Referral Hospital

**DOI:** 10.4314/ahs.v24i1.32

**Published:** 2024-03

**Authors:** Peterson Mulinde

**Affiliations:** Makerere University College of Computing and Information Sciences, Information Technology

**Keywords:** Healthcare technology, older persons, mobile phone

## Abstract

**Introduction:**

The global estimate of the aging population is progressively increasing in low and middle-income countries and this is accompanied by the limitations associated with the need for equitable and efficient healthcare delivery among this dire population. Unfortunately, despite the increasing numbers, the adoption of mobile phones is not balanced in the different populations with research showing young persons' adoption rate is higher than that of elderly persons.

**Objective:**

This current study was conducted to identify elderly people's perceptions of the use of mobile phones to support the self-management of long-term illnesses at Kiruddu National Referral Hospital.

**Methods:**

This descriptive-cross-sectional design study was conducted on a sample population of 30 elderly individuals older than 60 years admitted at the outpatient department of Kiruddu National Referral Hospital, Kampala, Uganda. We conducted face-to-face interviews following an interview guide and one focus group discussion. We later used a feature mobile phone and a tablet mobile phone to assess the individual ease of use of each device. The audio recordings were professionally transcribed and transcripts were coded into NVIVO version 12 analysis software for thematic analysis

**Results:**

Almost all of the respondents who visited the facility had an ailment that hindered their full utilization of the mobile phone to support their self-care. This together with other factors like financial constraints, lack of support from the health workers on how to use mobile phones to support health, inadequate support from the facility, and cost of mobile data among others.

**Background and Conclusion:**

This study provides empirical evidence that there is hardly a known mobile phone adoption model to enable policymakers, systems developers, and health workers to promote the elderly population's use of mobile phones to manage their long-term illnesses in Uganda.

## Introduction

Previous studies conducted worldwide indicated an increased number of the elderly people who are not using mobile devices, this is also favored by barriers that make adoption to these mobile devices difficult (Fletcher & Jensen, 2015). Mobile phones have been promoted for making phone calls, sending and receiving messages, and data. Before, a typical mobile phone was mainly used to make phone calls, but today, mobile phones' developers have expanded their capabilities to endless functions like accessing the internet, and used as remote controllers for other phones.

According to the Global System for Mobile Communications (GSMA) real-time intelligence data, mobile phones usage among people has highly increased, with the current statistics showing that 5.13 billion people now own mobile phones but despite the shooting numbers, adoption of mobile phones is not balanced in the different populations with research showing young persons' adoption rate is higher than the elderly persons (Jan & Mayuran, 2019).

Globally mobile phones adoption for self-management of the elderly outside hospital has not been fully examined in literature and yet, 8.1% of the world's population were older adults in 1960, and this number grew to 10% in 2000, with estimates suggesting that 21.4% of the population will be senior citizens in 2050 (Chiu & Liu, 2017; Whitehead & Seaton, 2016). While management of long-time illnesses is part of the intervention, they cannot help with lifestyle changes and real-time health monitoring. In the United States of America, technological interventions like smart homes, videophones and sensors suggest mobile device to improve management of health among the elderly (Joe & Demiris, 2013). In Italy, a mobile based wireless intervention helped to improve communication between medical teams and patients. The responses which were automatically sent to an authorized website showed that three of the four quarters of patients preferred use of mobile device to communicate with their care givers instead of physically visiting the hospital (Blake, 2008).

In the sub-Saharan Africa (SSA), elderly population is expected to increase to 67 million by 2025 alongside the social problems faced by this population like high poverty levels, remoteness, neglected by relatives and Non-Communicable Diseases (NCD) diseases (World Health Organisation, 2019). However, SSA's today's generations of older adults, especially in developing countries, have not grown up with Information and Communications Technologies (ICT) that are widely available these days making adoption to these mobile phones hard. This problem will probably not go away, as new technologies and their interfaces and interaction styles persistently increase. Thus, to make new technologies usable and useful for older adults, we need first to fully examine the complex patterns involved in technology (mobile phones') adoption and use among this population (Kim, Gajos, Muller, & Grosz, 2016). In Malawi, 75 Community Health Workers (CHWs) reported the effectiveness of a mobile intervention that included communication with their district referral hospitals. A Short Message Services (SMS) based communication and professional networking to support CHWs was conducted to assess the extent to which SMS capabilities facilitated and improved the quality of medical care that they provided (Betjeman, Soghoian, & Foran, 2013).

Uganda's elderly population (60 years) starts at 621,144 males and 809,43 females, and this number reduces with increase in age (Mundi, 2019; UBOS, 2019). The older persons national population projection by 2050 is expected to reach 5.4 million (Nzabona & Ntozi, 2015), which shows a high vulnerability to a great percentage of the country's population. The Uganda Ministry of Health (MoH) health sector Development plan 2015/16-2019/20 suggested that with the increasing life expectancy and the number of elderly persons increasing there is need to improve the quality of life in old age by preventing and treating diseases and disabilities in older adults. The ratio of patient to medical personnel in Uganda is also worsening, with numbers of clinical officers and enrolled nurses being 94% and 85% respectively (Lakuma *et al.*, 2016), yet most of the complications can be self-managed by the patients at home. The Sustainable Development Goal (SDG) three is aimed to ensure healthy lives and equal well-being of all populations regardless of age differences. This calls for assisting measures (mobile phones) to enable equitable health delivery across the country.

Elderly adults have comprised the fastest growing population adopting mobile phones over the past decade. However, how their experiences can shed light on self-management outside hospital has not been examined much in the literature (Chiu & Liu, 2017). Previous literature about the elderly people in Uganda mainly draws attention to poverty (Najjumba-Mulindwa, 2003), loneliness (Nzabona & Ntozi, 2015), remoteness or distance from the health center (Maniragaba et al., 2019), NCD's (Wandera, Ntozi, & Kwagala, 2014), chronic obstructive pulmonary diseases (COPD's) (Siddharthan *et al.*, 2019), and physical movement (Nankwanga, 2012). Despite all the research done in Uganda, data on mobile phones adoption for self-management of the elderly outside hospital is still scanty mainly because these studies were carried out long time back and also, they were covering other different areas but not mobile phones. Hence, current compact reference works for mobile phones adoption and elderly health care management outside the hospital is not available.

## Materials and methods

### Study design

The study was a descriptive cross-sectional and design science methodology was used. Face-to-face interviews were conducted on the elderly persons who had visited the facility and had been enrolled to the Outpatient Department. All necessary authorizations were obtained to ensure this study can be implemented in this particular setting.

### Study setting

This study was carried out at Kiruddu National Referral Hospital which operates under the Ministry of Health.

Located in Makindye division, Kiruddu National Referral Hospital has a bed capacity of 200 plus its recorded health worker capacity of 100. Due to the ongoing renovations that were happening at Mulago national referral hospital, Mulago hospital transferred 14 of her clinics to Kiruddu National Referral Hospital and these include; hypertension, thyroid, diabetes, kidneys, heart problems, infectious diseases, skin problems, diseases of the brain, lungs and burns.

### Study population

The target population for this study was older persons aged 60 years and above admitted at the outpatient Department at Kiruddu National Referral Hospital.

### Eligibility measures

#### Inclusion measures

Every elderly person who consented and accepted to participate in the study.

All elderly people who are 60 years and above.

#### Exclusion measures

All elderly persons who declined to give their consent of participation.

Very ill patients were not recruited to this study.

### Sampling

The researcher used purposive sampling. This is commonly used as a qualitative method because it is the most successful when data review and analysis are done in conjunction with data collection (Mack, 2005).

### Quality control

All research assistants enrolled to assist in the data collection were trained on how to approach older persons, how to interview them using both the recorders and the interview guides.

All audios were cross-checked at the end of each day for completeness and recorders were stored securely.

### Ethical consideration

The researcher got ethical approval from Makerere University College of Computing and Informatics Sciences (MakCoCIS) under the department of Information Technology. We also acquired approval from the Mulago hospital Research and Ethics board. We acquired administration clearance from the Kiruddu National Referral Hospital research office. We also got consent from each participant before being included in the study.

### Study tools

As a requirement of the authorities, all tools must be converted to the local language where data collection is to be conducted and therefore the interview guide was administered both in English and Luganda because these are the common languages used by people located in the central region of Uganda.

### Data collection

Data was collected from participants between July 2021 and August 2021. Two nurses were employed and trained on how to identify the participants who the study wanted. The nurses periodically identified the potential participants and were enrolled to the study. A total of 30 participants was enrolled to the study which included one FGD of 8 elderly people and 22 interviews for the elderly.

### Data analysis

The collected raw data was manually cleaned, coded into, and analyzed using NVIVO version 12. The output was generated as a thematic report in form of participant responses.

Older persons were categorized into three parts, those who own smart phones, those who own Feature phones and those who do not own a mobile phone. Variables were computed as predictors to mobile phone adoption.

## Results

### Health seeking practice among elderly

#### Reasons for seeking Health care

The main health complaint reported by majority of respondents was heart problems and hypertension. Some respondents said they come to the hospital to seek treatment and medical examination, for HIV/AIDS, backache, blood clot in the leg, dizziness and Seek ENT services:

*“Am a pressure patient and they have given letters to go for the heart check-up because there is when my heart beats very first like someone who has committed a crime or seen something big. So, I have been told to go for the heart check-up but the money I have is not enough according to what they have told me”*. (FGD1, female)

### Perception about attitude and experience of doctors who care for the elderly

#### Positive attitude

Majority of respondents reported Positive attitude towards doctors that cared for them. They said doctors created good rapport with elderly and encouraged them to adhere to medication:

*“They really take care of us, when i came here for admission; they cared for us so well. I have not gotten any problem with them as a new patient…my sugar levels problems were identified at city council hospital and they referred here…, People said doctors at Kiruddu doctors are bad, they kill patients. I said, I am in pain, if I am to die let me die, but when I reached here, I did not see of whatever they were saying they handle us well”*. (Individual interview 15, 63 years, female)

### Perception about doctor's experience in technology

Some of the respondents said doctors had experience in technology and rated them (10/10). This is because they made right diagnosis using technological machines for example heart scan and checked for rightful health information through mobile phones, *“according to the technology they use me they took me to the scanning machines to scan the heart”* (FGD1, female)

Doctors monitored patient's progress through technology:

*“Doctors follow information given to them given to them; let us say like this has come on the phone. The instruction they have to follow… the doctor has to weigh my progress and it seems he uses the phone properly because it shows him the progress”* (Individual interview 6, 67 years, male)

Doctors used technology to make rightful diagnosis:

*“Because they don't guess when they are treating you, they first find out what you're suffering from, taking your blood sample and conform that's the disease you are suffering from and also sometimes whenever they tell you to go for a certain check-up you find out that it is the cause of the disease”* (FGD1, female)

Doctors checked medicine on phone:

*“The doctors, I see everyone has a big phone whenever they are working on us, I see them checking for medicine on their phones sometimes which means they understand them. I think that out of 10 we can give them 7/10”*. (Individual interview 2, 62 years, female)

### Negative attitude towards doctors

The majority of respondents who reported negative attitudes towards care received from doctors said they did not receive support from health doctors on how to use phones:

*“But for the phone I hadn't got any person telling me on how to use it, but what I see on my side there some people that call me and they take long on the call but by the time it switches off it has heated. I don't feel proper in the ears but I have never got any one telling me that don't receive it near the ear”*. (FGD1, female)

Whereas some respondents said, most doctors are trained to care for elderly people, *“I don't doubt most doctors are well trained to help the elderly people”* (Individual interview 10, 68 years, male), one said not all of health officials are well trained to take care of old people.

Some respondents said some doctors were not ready to receive old people, *“the doctor might back at you, not explain very well about is going on, I want when I reach to doctor and as it's my life and what you studied and we discuss”* (Individual interview 14, 69 years, female). Some respondents said they experienced poor customer care and labeled doctors as harsh, *“… some of them are harsh but we have nothing to do we have to pay attention on what they are telling us”*. (Individual interview 12, 64 years, female).

*“The doctors of long ago had customer care: may be because many were taught by the whites, I even have my friend who is doctor, when he sees the doctors of today says they taught us like this. What I see, what they study professional, I think is enough but handling people, they reduced its strength but than the previous ones”* (Individual interview 14, 69 years, female)

### Behavioral intention of use of mobile phone

#### Previous experience with use of mobile phone for health purpose

Majority of respondents possessed an ordinary button phone or a smart mobile phone with own WhatsApp. Commonly used types of phones among the elderly included; Huawei, itel. Duration with phone was more than two years. *“I have been having a phone, when children decide they send me a phone like Huawei, they can send me itel, they can send me any phone, even that one I never expected”* (Individual interview 3, 60 years, male).

### Components or features of mobile phone commonly desired and used by elderly

Making and receiving phone calls and use of a torch when power is off, *“If working torch on phone, I use phone menu button to put on torch and I can make call”* (Individual interview 7, 64 years, female)

Use of mobile money app where to receive or send money via phone, *“and when the children send me money they call, that mummy money has come have you seen it? and I say Yes, I have seen it thank you very much”* (Individual interview 13, 75 years, female)

Use of Radio, *“the radio is there but without earphones it does not work”* (Individual interview 15, 63 years, female)

Access calculator during business transactions, *“On my phone, I usually talk to my people, in most cases my children and my wife, even sometimes when I need to make some calculations, when I am using the phone, I use the calculator of the phone”* (Individual interview 3, 60 years, male)

Receives phone calls messages from doctor advising on what to do or from friends, workmates and family, *“I receive messages telling me who has died in the area or sometimes if someone sends me some money and another thing, the Radio and calling other people that I want”* (Individual interview 12, 64 years, female)

### Components of mobile phone not commonly used

One elder said did not commonly use phone pads (earphones), *“I see earbuds with most youths but to me am saying that if I don't put the phone near the ears and put those wont the be working”* (FGD1, female).

### Phone technological acceptance factors

#### Advantages of using mobile phone towards health of elderly people

##### Financial use of mobile phones

Some respondents mainly from FGD said they save money and transact business, *“I save money on phone, I also sell goods on it like soap I put on the network, my friends call and tell I need a jerry can so the phone develops me”* (FGD1, female). Received or sent money via mobile money, *“I used it to receive phones and also me to call and also to receive money from my daughters that have sent me money for treatment and personal issues”* (FGD1, female).

Withdraw and save Mobile money, which saves time due to limited bank movement, *“You may also get your money and decide to keep it on phone and where there is need you just go and withdraw the money”* (FGD1, female).

Use phone to borrow airtime or send school fees, transport to students and pay for other utilities via the phone:

*“So, the phone helps us a lot, children school fees as where the child is sent back home for school fees, they just call the parents and inform them that I have been sent even when I have been in the village and the children are in Kampala, I have been sending the school fees via the phone. Even when they get a problem they inform also when they get their holidays, they call me and I send them transport to bring them to the village”* (FGD1, female)

##### Psychosocial support

Majority of respondents said the main advantage of use of mobile phone was to Communicate with people (receive and make calls). They used Mobile phone to call children and other people, in case they needed some personal things items or to communicate sickness, *“Call relatives (wife, children) for social support when sick”* (Individual interview 1, 70 years, female). Most respondents said they obtained from relatives support for example, financial to seek medical health or food for better health, using mobile phone, *“I call the children, like now I called them and they sent money to bring me here, because the person who has brought me here is to be paid…”* (Individual interview 13, 75 years, female).

Some respondents said they read mobile messages that are found encouraging such as health messages, *“… they might tell you that to avoid diabetes you can do this, those messages are very useful”* (Individual interview 14, 69 years, female)

##### Stress management

Majority of respondents said phones enhanced communication to other people, which reduced stress and anxiety:

*“… I only see good things about phones because you can communicate to your people in far distances. you can tell someone to give you like shs. 1,000 or put for you airtime and call someone you have heard that has got a problem to establish if is true, they are dead or alive”* (Individual interview 3, 60 years, male).

Phone takes away boredom when listen to music, or when children help to play phone games, *“reading WhatsApp messages and playing super three games on phone on takes away boredom and you laugh”* (Individual interview 1, 70 years, female)

Access radio as source of news, *“the radio quickens and helps us to know what's going on in world like the news, etc, such that your able to leave in the country that you and understand or know the news in the country in that day”*. (FGD1, female)

One respondent uses phone to time sleep to avoid missing an important appointment:

*“It helps me because even at night I sleep when it's close to me, I use it to know the time, still to know if it has come to daytime, I get to see that it would not have to miss on someone and it should be there when it is helping you”*. (Individual interview 1, 70 years, female)

### Perceived benefits and mechanisms of using mobile phone for health purpose

Majority of respondents said mobile phone App had potential to offer health solutions by teaching about Health. Doctors could send medication prescription to patients through phone.

Seek medical advice for self and others through phone especially during peak of COVID-19, *“I am here another patient is in the village it helps me to know how those people are doing if they are fine or not”* (Individual interview 7, 64 years, female)

*“Like we know using them or you call and communicate with people like as we are in the Covid pandemic use such medication for example mango leaves cook and steam yourselves that is to say you get a lot of information that helps your life or your friends' life and you also inform others”* (FGD1, female)

*“You call and they tell go to the pharmacy or reads you the medication and you buy such that you treat yourself or a child. Like we know… as we are in the Covid pandemic use such medication for example mango leaves cook and steam yourselves that is to say you get a lot of information that helps your life”* (FGD1, female)

Some respondents said phones were a source of health information during peak of COVID-19. Some received health messages on how to live healthy by preventing COVID-19 through hand washing, wearing a mask and social distancing, *“now covid-19 messages they come to tell a person to avoid/protect from the disease, don't touch hands, you have to wash your hands everywhere you go, wear a mask”* (Individual interview 5, 60 years, male).

Consulted doctor using phone, *“I call some and I tell them I slept badly, my back is paining and they tell you what to do”* (Individual interview 12, 64 years, female). Some respondents said they called doctors to explain about health or diseases to seek referral advice, *“…I call and ask that am suffering from this what should I do and they direct me that go here or go to Kiruddu or there those at Kampala Road and anywhere else”* (FGD1, female).

Some respondents said they liked the appointment reminder calls:

*“Where I receive medicine from, the doctors call me one day before the date for reporting to the hospital and tells me Rose tomorrow I need you. Now the doctor takes the responsibility and reminds me like he does not want me to forget and be like doctor I forgot the doctor reminds me and I also know that tomorrow early morning I have to be in the hospital.”* (Individual interview 7, 64 years, female)

*“The good thing is that after giving you the medication they give you a particular date that you have to report back and they treat you again. Now for the treatment they do care. When they give a date, they check on you again and see how you are or whether you have any change. If there is no change then the try a different medication and when we leave our contacts, they call us and tell you that we need you on such and such date”* (FGD1, female)

Use phone in own treatment to enhance adherence and communicate to people during COVID-19:

*“My phone helps me a lot especially in this Covid pandemic, you call ask about the family and ask them how they are and they also ask how you are. In the village I have grandchildren and also inform you that your grand child is such and such condition so the phone helps me such situations and where I can't reach it's able to help me reach there”*. (FGD1, female).

Receive counselling about health-related issues, follow up test results and check on sick relatives:

*“Now on my phone it normally helps me on the sick people I normally have seek people let's say I may be in Kampala and the call me that in the village there is a sick person that the headache has worsened come and we take him to the hospital. Then we call the doctor and informs us which hospital can we go to and as we reach, they check the headache and the send us the message that the results are out”* (FGD1, female)

Conduct Community mobilization for immunization through phone:

*“I usually call, like in my village I work as a VHT (Village Health Team). so, I inform the doctors at the division or our coordinator that our village has got such and such disease outbreak. I call the doctors there is a center, I mobiles to immunize children so I ask them whether they are coming or not and inform them to come with medication for the immunization of virginal cancer and also call so parents that I know informing them about immunization”*. (FGD1, female)

### Facilitators or requirements for use of mobile phone adoption Model for the elderly

#### Infrastructure or functionality of phone

Majority of the respondents said having an internet infrastructure to ease accessibility of app and use of a smart phone and availability of data: *“If older persons could have tablet phones that is better”* (Individual interview 10, 68 years, male).

### Challenges, disadvantages (risks, dislikes, negative effects, and limitations) of mobile phone use for elderly people

#### System or infrastructure related

Majority of respondents were inconvenienced by low or dead phone battery. *“First there is the phone battery you have just used it a little and you see that the system it dead, you find that you can no longer charge it. You have to look for a place where they charge batteries, so also the battery disturbs me a lot”* (FGD1, female)

Poor network penetration was a major problem reported. Especially for MTN network that was ever off, *“network, especially that of MTN is ever off I need to first go out, sometimes I get it in the house that's how the network treats me”* (FGD1, female). Poor network connectivity (on and off) was reported especially in rural areas, *“network problem exists, maybe when am in the village but when I am here it is not that much poor”* (Individual interview 7, 64 years, female).

Lack of data hinders phone messages from coming through. When Sim cards fail, they are replaced but it becomes difficult to retrieve phone numbers:

*“The phone had not disturbed me apart from may be the sim card was forced and it could work. When I went to MTN and they changed for to another Sim card, it worked properly. What disturbed me when they changed the Sim card my numbers could not come properly, I failed to find my numbers whereby there were some which were they and some which and some which were not there, that's what has disturbed a lot so far”* (Individual interview 14, 69 years, female).

### Security

Some respondents were concerned about confidentiality and safety of mobile phones because phones often Stolen and therefore need to be protected from thieves and traced if stolen, *“so, when a phone is stolen, we should know how to get the thief by tracing it”* (Individual interview 10, 68 years, male)

Majority of the respondents were concerned about mobile money conmen; they were curious to know about how fraudsters got their contacts and they think get from mobile telecom. *“I don't want they also steal my money sometimes; you leave your money and after remembering, you find that there isn't anything”* (Individual interview 13, 75 years, female)

*“He calls you and asks what the last digit is of you pin and yet he has told you that don't speak it and he is viewing it and you tell him let's say the last digits are forty and tells you it's not the one. That means these telecom companies don't keep our things properly and that my major problem, I want the pin code to be invisible to people”* (FGD1, female)

*“What surprises me are these corn men who call us, and I wonder because they call you by your own name and I wonder where they get my number from, they are able to access my number or my names. I think the manufacturers the ones who manufacture them if they can put something so that a person cannot easily call you like a thief”* (Individual interview 2, 62 years, female)

### Affordability

Most of the respondents were concerned about expensive cost of tablet. They wondered about the cost of the app as well Cost of app, whether it would be free access or not. They said smartphones or tablets were expensive for elderly, *“… do I have that money to buy another phone I don't have, apart from when the children give me and something else, I don't want slide”*. Cost of internet and data is expensive yet elderly have financial constraints.

### Individual challenges

Some respondents said Poor eyesight hindered their phone usage. They could not read mobile phone message due to poor eyesight. This caused failure to view some phone features:

*“Right now, I have a button phone. I would have wanted a touch but may be because of the eye problem… my eyes, as my eyes were affected by insulin, I don't see well … I see the buttons but when it comes to seeing time or date, I don't see them properly or the phone when I want to call someone”* (Individual interview 15, 63 years, female )

Few respondents disliked phone bright light upon switching on phone. They disliked torch light because it destroyed eyes. *“For me the phone light normally disturbs me, like especially during day I need to get a shade where I can use it properly, it's light. Even hearing I need to be put in a higher volume”* (FGD1, female)

Hearing impairment. Poor hearing was a barrier reported by respondents, phone usage required high volume on phone for such to use, *“since that my ears don't hear properly sometimes when someone is near me, I just give him the phone to listen and I ask him what the other person has said”* (Individual interview 12, 64 years, female):

*“I speak properly but there is when it cuts off the voice but there is when I speak put when put to when I stop and I have spoken properly. When the network is lost and I cannot hear properly but mainly is the voice for it, it's so low. Then I increase to the maximum but again I don't hear properly”*. (FGD1, female)

Some respondents found difficulty to press security code due to sickness, *“I am the only one who has to know that here I press two or three or four, it might be difficult for me, because sometimes I may be sick”* (Individual interview 7, 64 years, female)

One respondent disliked funny noises from phone, *“the battery works properly but there are some funy noises it makes which I don't like”* (FGD1, female)

### Health provider or facility related

There was concern about some health providers not contacting patients yet had patient's contact sheets:

*“I would also ask that because they collect our contacts and they have them but I have never got any message from Kiruddu informing us about anything expect for a doctor that you have his contact and you contact him. But for that they should revise it such that they are able to inform us and also let us know what we are supposed to do with the various medication that either take it in the morning or evening, they should revise that”*. (FGD1, female)

### Knowledge gap

Majority of respondents said they did not know about the existence of mobile health app.

Some respondents do not know how to read and write: *“Like me who doesn't know how to read… They explain to me but I don't understand. I have a neighbour, she has challenges with Luganda because she only knows English only and reading Luganda challenges her, when they send a message in English there, she can explain for me but she does not know Luganda”*. (Individual interview 15, 63 years, female)

## Discussion

The Poor functionality of phone system was evidenced among several respondents and yet proper functionality of a technology device is an important factor in determining adoption by an individual. In studies conducted by Rapudo (2013) and Yang (2022) show functionality of a technology or mobile device is also a determinant of adoption despite the fact that it is always not observed (Rapudo, 2013; Yang *et al.*, 2022).

Results from the interviews also revealed that poor network penetration of some service providers was not effective in some areas hence resulting in poor usage of mobile devices by the elderly people. There is clear evidence that some network companies in Uganda lack network coverage in some parts of the country (Bambino, 2015).

Like any other open platform, mobile devices also have the same limitation of being flooded with unwanted information that is directed to every mobile device user regardless of the age. Irrelevant information defines greatly the adoption of a particular technology or device. This is another ignored field where the user of a mobile device is not given a chance to identify particular information relevant to their needs in order to influence their level of adoption.

Due to their failing health, elderly people also have challenges with the audio of the mobile devices during a call. This has not been identified by researchers as a major challenge mainly because it differs among the elderly, meaning that it is a challenge to only a few.

Lack of data was also identified as a hindrance mainly because today day-to-day information and business runs on an internet enabled phone. This challenge is also in line with Hayes (2019) study proves that the lack of internet connection on a mobile device affects an individual's usage of a phone (Hayes *et al.*, 2019).

Security was observed as a challenge and this was divided into two categories; physical security and information security. Being a broad field, this study only found out that respondents found physical security a big concern in Uganda. Mobile phones have been the leading target of thieves in the country. This is accompanied by related crimes of mobile money and bank account details (Bagala, 2022).

The cost or affordability was also identified and divided into three categories; ability to buy a mobile phone, ability to buy an application and ability to afford mobile call tariffs. All these are determinants of a person's adoption rate. A paper by Muhalia (2010) highlighted that affordability aspect is always left out when determining the factors affecting adoption yet it is very important. This is due to the difficulties related to differential tariff pricing and other related worries (Muhalia, 2010).

This study also discovered that individual challenges like poor eye sight determines the adoption of mobile phones among the elderly. This challenge is in line with a challenge identified by a Wang's (2020) study which showed that visual impairment among its participants was the main determinant of low adoption to mobile devices (Wang et al., 2020).

There was a challenge of pressing the buttons by the participants in this study mainly because of their related illnesses. Navabi's (2016) research clearly defines this problem, however there is no clear reason why they had difficulty in pressing the buttons in Navabi's research (Navabi *et al.*, 2016).

Majority of researchers keep ignoring the knowledge gap as a determinant to adoption of mobile phone among the elderly and yet it keeps coming up during the identification of challenges hindering adoption. This study identified that the limited knowledge about the existing mobile applications which elderly people can use to manage their long-term illnesses is also limiting their adoption of mobile devices.

This research also identified that limited engagement of healthcare facilities in mobile health also limits the adoption of mobile phones by the elderly. This has created a division between technology and health facilities where the elderly seek medical care for their long-term illnesses (Shahbazi, Bagherian, Sattari, & Saghaeiannejad-Isfahani, 2021).

## Conclusion and recommendation

There is hardly a known mobile phones adoption model to enable policy makers, systems developers and health workers promote the elderly population use mobile phones to manage their long-term illnesses in Uganda.

The ministry of health is the top most body which governs all government national referral hospitals in Uganda, therefore government should establish supportive policies in order to improve the mobile device acceptance by referral hospitals. This will can enable the healthcare professionals to take it up, hence enforcing it to the patients resulting into convenience during service delivery.

The ministry should also invest in the creation of innovative endeavours for mobile device awareness in order to enable the public understand the benefits of using mobile devices to manage their long-term illnesses rather than lining up in cues at the hospital.
